# Evaluation of the Safety of Calcitonin Gene-Related Peptide Antagonists for Migraine Treatment Among Adults With Raynaud Phenomenon

**DOI:** 10.1001/jamanetworkopen.2021.7934

**Published:** 2021-04-19

**Authors:** Ilana D. Breen, Caitlin M. Brumfiel, Meera H. Patel, Richard J. Butterfield, Juliana H. VanderPluym, Leroy Griffing, Mark R. Pittelkow, Aaron R. Mangold

**Affiliations:** 1 Department of Dermatology, Mayo Clinic Arizona, Scottsdale; 2 Department of Biostatistics, Mayo Clinic Arizona, Scottsdale; 3 Department of Neurology, Mayo Clinic Arizona, Phoenix; 4 Department of Rheumatology, Mayo Clinic Arizona, Phoenix

## Abstract

**Importance:**

Calcitonin gene-related peptide (CGRP) antagonists have demonstrated tremendous promise in migraine management. However, these medications decrease reflex vasodilatory response, which may lead to exacerbation of microvascular disease in susceptible patients, such as patients with Raynaud phenomenon (RP).

**Objective:**

To investigate the microvascular complications of CGRP antagonists in patients with underlying RP.

**Design, Setting, and Participants:**

This retrospective cohort study was performed from May 18, 2018, to September 15, 2020, in Mayo Clinic Health System patients with Raynaud phenomenon while undergoing CGRP antagonist therapy to treat migraine. Inclusion criteria were age older than 18 years, history of migraine, past or current treatment with CGRP antagonists, and diagnosis of primary or secondary RP.

**Exposure:**

Treatment with CGRP antagonists.

**Main Outcomes and Measures:**

The main outcome measure was microvascular complications (eg, worsening RP, digital ulcerations, and gangrenous necrosis) after initiation of treatment with a CGRP antagonist. Patient demographic and clinical characteristics were compared between those who experienced complications and those who did not.

**Results:**

A total of 169 patients (163 [96.4%] female; 151 [89.3%] non-Hispanic White; mean [SD] age, 46 [13] years) were identified. Of the 169 patients, 9 (5.3%) exhibited microvascular complications, ranging from worsening RP to gangrene and autonecrosis that required distal digit amputation. Comparative analysis did not find statistically significant differences in demographic or clinical characteristics between the 2 cohorts. All 9 patients with complications were female (mean [SD] age, 40 [12] years). Five of the 9 patients (55.6%) had previously diagnosed RP; in 3 the RP was primary, and 2 it was secondary to scleroderma. The other 4 patients (44.4%) were newly diagnosed with RP. Eight of the 9 patients (88.9%) had chronic migraine; 4 had migraine with aura, and 5 had migraine without aura. The CGRP antagonist agents temporally associated with the microvascular complications included galcanezumab (in 3 patients), erenumab (in 5 patients), and fremanezumab (in 1 patient).

**Conclusions and Relevance:**

The results of this study indicate that microvascular complications of CGRP antagonist use in patients with underlying RP are uncommon. The incidence of serious adverse events, although rare, warrant caution when considering the use of these agents in patients with RP.

## Key Points

**Question:**

In patients with Raynaud phenomenon, what is the microvascular complication risk of calcitonin gene-related peptide (CGRP) antagonist use?

**Findings:**

In this cohort study of 169 adults with Raynaud phenomenon, 9 patients had microvascular complications after CGRP antagonist use. Two of the 9 patients had severe adverse events, including digital autonecrosis that required distal amputation.

**Meaning:**

This study suggests that microvascular complications are uncommon in patients with Raynaud phenomenon who are taking CGRP antagonists; however, the incidence of adverse microvascular events with high morbidity warrants caution in prescribing CGRP antagonists in these patients.

## Teaser

This cohort study investigates microvascular complications associated with calcitonin gene-related peptide (CGRP) antagonists in patients with underlying Raynaud phenomenon.

## Introduction

A new class of medications known as calcitonin gene-related peptide (CGRP) inhibitors has been recently approved for the treatment of episodic and chronic migraine. There are 2 types of available CGRP inhibitors: (1) monoclonal antibodies that bind the CGRP receptor or ligand and (2) small-molecule CGRP receptor antagonists. These medications suppress activity of CGRP, a neuropeptide located in the peripheral and central nervous system that is involved in pain modulation, particularly in the trigeminovascular system.

In addition to its role in the nervous system, CGRP confers potent vasodilatory effects, which may clinically manifest as flushing.^1^ Deficiency of CGRP may play a crucial role in the pathophysiology of scleroderma and Raynaud phenomenon (RP). In scleroderma, there are fewer CGRP-supplying nerves, and in RP, there may be CGRP deficiency in the distal or acral skin.^2,3^ Cutaneous blood flow increases in these patients after CGRP infusion.^3,4^ A previous study^5^ examined the effects of systemic therapy with calcitonin in patients with scleroderma, finding a reduction of digital ulceration and improvement in pulmonary function after infusion. In another study, the use of CGRP antagonists was associated with digital ulceration in 2 patients with underlying RP.^6^

Microvascular complications of migraine therapies predate the advent of CGRP modulators, as evidenced by worsening RP documented in the use of vasoactive medications, such as ergot alkaloids, triptans, and β-blockers. With the emergence of CGRP antagonists as a mainstay in prophylactic and rescue migraine therapy, it is crucial to identify patients at risk for complications to develop appropriate and safe prescribing guidelines. In this study, we identified patients with primary or secondary RP while taking CGRP antagonists for migraine therapy and assessed for cutaneous microvascular complications.

## Methods

This retrospective cohort study was performed with a medical record review conducted through the Mayo Clinic Research Database from May 18, 2018 (after US Food and Drug Administration approval of first CGRP medication), to September 15, 2020, to identify patients of all ages with underlying RP (primary or secondary) who were prescribed CGRP antagonists for migraine. Approval was obtained from the Mayo Clinic Institutional Review Board, and a waiver of informed consent was granted. All data were pooled and deidentified for analysis. This study followed the Strengthening the Reporting of Observational Studies in Epidemiology (STROBE) reporting guideline.

An initial search of all patients in the Mayo Clinic Health System (Minnesota, Arizona, and Florida) with *International Statistical Classification of Diseases and Related Health Problems, Tenth Revision (ICD-10)* codes related to RP and scleroderma was performed, which yielded a total of 29 422 unique patients. We then globally searched all the clinical documents of the captured patients for any of the following terms: *erenumab*, *fremanezumab*, *galcanezumab*, *eptinezumab*, *ubrogepant*, *rimegepant*, *Aimovig*, *Ajovy*, *Emgality*, *Vyepti*, *Ubrelvy*, *Nurtec*, *CGRP*, and *calcitonin gene-related peptide*. This search returned 274 unique patients. Each medical record was individually reviewed for migraine history, RP and rheumatologic history, CGRP antagonist prescription history, and notation of microvascular complications (eg, new-onset RP, worsening frequency or severity of existing RP, ulceration, and gangrenous necrosis) anywhere in the clinical documentation. Patients were included if they met the following criteria: history of migraine diagnosis of primary or secondary RP and past or current exposure to CGRP antagonists. A total of 169 patients met the inclusion criteria. Data abstracted included the following: age, sex, race/ethnicity, migraine history (eg, type or presence of aura), migraine medication history (eg, monoclonal antibody vs small-molecule CGRP antagonists), rheumatologic history, RP risk factor history (eg, anatomical, vascular, environmental, pharmacologic, rheumatologic, hematologic, or infectious conditions^2^), and pertinent associated complication history.

Patient demographic and clinical characteristics, including risk factors for RP, were compared between those who experienced complications and those who did not using the Kruskal-Wallis test or Fisher exact test, where appropriate. Similarly, underlying CGRP medication use was compared between groups using the Fisher exact test. All hypotheses were 2-sided with *P* < .05 considered statistically significant. Analyses were performed in SAS statistical software, version 9.4 (SAS Institute Inc).

## Results

**Figure 1.**
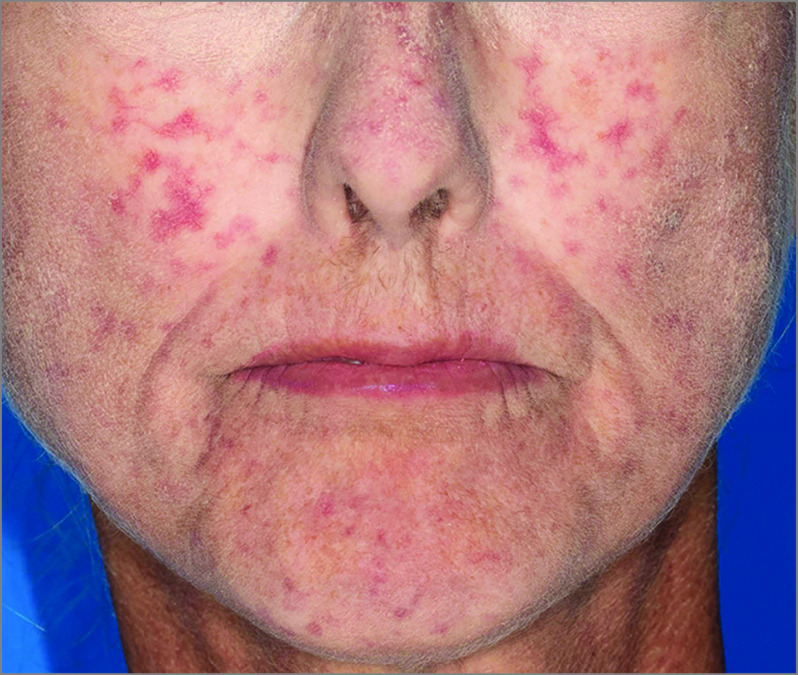
Worsening Facial Telangiectasias in a Patient With Scleroderma After Use of Galcanezumab

**Figure 2.**
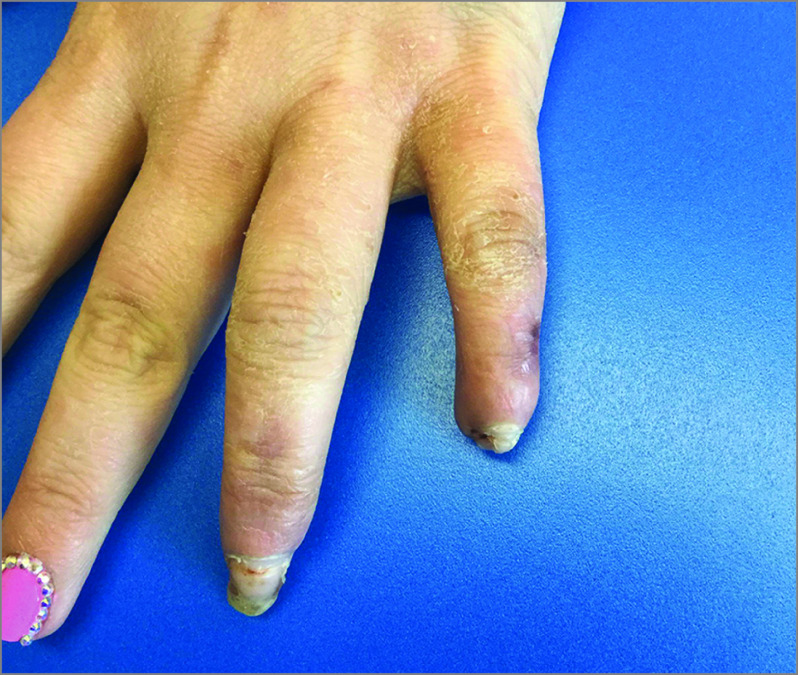
Digital Necrosis With Subsequent Autonecrosis in a Patient With Primary Raynaud Phenomenon After Use of Erenumab Image was taken 30 days after amputation of left distal fourth and fifth distal fingertips.

A total of 169 patients (163 [96.4%] female; 151 [89.3%] non-Hispanic White; mean [SD] age, 46 [13] years) were identified. Demographic characteristics, presence of established RP risk factors, and migraine history among the entire cohort are detailed in Table 1. Of the 169 patients, 9 (5.3%) exhibited microvascular complications after initiation of CGRP antagonist therapy for migraine. Comparative analysis of demographic and clinical characteristics, including migraine history and RP risk factor history, between the 2 patient cohorts (patients with complications and those without complications) was performed (Table 1). All 9 patients with complications were female (median [interquartile range] age, 40 [12] years). Seven of the 9 (77.8%) were non-Hispanic White and 2 of 9 (22.2%) were Hispanic. Their microvascular complications are summarized in Table 2, along with other pertinent demographic and clinical characteristics. The complications ranged from worsening RP (characterized by more frequent episodes of pain and discoloration elicited by cold temperature exposure) to worsening facial telangiectasias (Figure 1) to digital gangrene and autonecrosis that required distal digit amputation (Figure 2*)*. Five of the 9 patients (55.6%) had previously diagnosed RP; in 3 patients, RP was primary, and in 2 it was secondary to scleroderma. The other 4 patients (44.4%) were newly diagnosed with RP after administration of CGRP antagonists. These patients did not undergo any immunologic testing or evaluation with a rheumatologist for RP. Eight of 9 patients (88.9%) had chronic migraine; 4 had migraine with aura (mostly visual), and 5 had migraine without aura. The CGRP antagonist agents temporally associated with the microvascular complications included galcanezumab (in 3 patients), erenumab (in 5 patients), and fremanezumab (in 1 patient). Several important confounders were identified, including coincident administration of potential exacerbating medication (β-blockers^7^), hematoma overlying the digit associated with RPs, and symptomatic worsening during wintertime. The mean time from CGRP antagonist induction to microvascular complication noted in the patients’ medical records was 163 days (range, 26-365 days).

**Table 1.** Summary of Baseline Demographic and Clinical Characteristics of the Cohort^a^

Characteristic	Cohort with complications (n = 9)	Cohort without complications (n = 160)	Total cohort (N = 169)
**Demographic characteristics**			
Sex			
Male	0	6 (3.8)	6 (3.6)
Female	9 (100.0)	154 (96.3)	163 (96.4)
Age, mean (SD), y	40 (12)	46 (14)	46 (13)
Race/ethnicity			
Non-Hispanic White	7 (77.8)	144 (90.0)	151 (89.3)
Non-Hispanic Black	0	4 (2.5)	4 (2.4)
Hispanic or Latino	2 (22.2)	5 (3.1)	7 (4.1)
Non-Hispanic Asian	0	3 (1.9)	3 (1.8)
Other	0	4 (2.5)	4 (2.4)
**Raynaud phenomenon risk factors**			
History of connective tissue disease	4 (44.4)	47 (29.4)	51 (30.2)
Scleroderma	2 (22.2)	10 (6.3)	12 (7.1)
Morphea	0	6 (3.8)	6 (3.6)
Rheumatoid arthritis	0	7 (4.4)	7 (4.1)
Sjögren syndrome	0	9 (5.6)	9 (5.3)
Behçet syndrome	0	1 (0.6)	1 (0.6)
Systemic lupus erythematosus	0	9 (5.6)	9 (5.3)
Dermatomyositis	1 (11.1)	3 (1.9)	4 (2.4)
Vasculitis	0	0	0
Mixed connective tissue disease	0	0	0
Paraneoplastic pemphigus	1 (11.1)	0	1 (0.6)
NOS or undifferentiated	1 (11.1)	9 (5.6)	10 (5.9)
Smoking status			
Current	0	7 (4.4)	7 (4.1)
Former	0	31 (19.4)	31 (18.3)
Never	9 (100.0)	122 (76.3)	131 (77.5)
Vascular risk factors or disease	3 (33.3)	82 (51.3)	85 (50.3)
Hypertension	3 (33.3)	36 (22.5)	39 (23.1)
Hyperlipidemia	1 (11.1)	61 (38.1)	62 (36.7)
Atherosclerosis	0	8 (5.0)	8 (4.7)
Peripheral vascular disease	0	3 (1.9)	3 (1.8)
Coronary artery disease	0	6 (3.8)	6 (3.6)
Diabetes type 2	0	2 (1.3)	2 (1.2)
History of medication use associated with RP	9 (100.0)	155 (96.9)	164 (97.0)
Amphetamines	2 (22.2)	24 (15.0)	26 (15.4)
β-Adrenergic blockers	6 (66.7)	88 (55.0)	94 (55.6)
Ergots	0	21 (13.1)	21 (12.4)
Interferon alfa	0	2 (1.3)	2 (1.2)
Triptans	9 (100.0)	147 (91.9)	156 (92.3)
Cyclosporine	0	1 (0.6)	1 (0.6)
Hematologic syndromes	0	5 (3.1)	5 (3.0)
Paraproteinemia	0	1 (0.6)	1 (0.6)
Cryoglobulinemia	0	1 (0.6)	1 (0.6)
Factor V Leiden	0	3 (1.9)	3 (1.8)
Environmental history			
Frostbite	0	2 (1.3)	2 (1.2)
History of anatomical variations	1 (11.1)	35 (21.9)	36 (21.3)
Thoracic outlet syndrome	0	3 (1.9)	3 (1.8)
Carpal tunnel syndrome	1 (11.1)	33 (20.6)	34 (20.1)
History of infectious diseases	0	8 (5.0)	8 (4.7)
Hepatitis C	0	1 (0.6)	1 (0.6)
Parvovirus 19	0	7 (4.4)	7 (4.1)
**Migraine history**			
Type (episodic vs chronic)			
Chronic	8 (88.9)	118 (73.8)	126 (74.6)
Episodic	1 (11.1)	14 (8.8)	15 (8.9)
Unknown	0	28 (17.5)	28 (16.6)
Presence of aura (n = 159)	4 (44.4)	70 (46.7)	74 (43.8)
Visual	3 (33.3)	49 (32.7)	52 (30.8)
Speech	0	3 (2.0)	3 (1.9)
Sensory	0	4 (2.7)	4 (2.5)
Brainstem	0	3 (2.0)	3 (1.9)
Vestibular	0	0 (0.0)	0 (0.0)
Aura, not specified	1 (11.1)	18 (12.0)	19 (11.9)
**CGRP antagonist history**			
Monoclonal antibodies			
Galcanezumab	3 (33.3)	76 (47.5)	79 (46.7)
Fremanezumab	1 (11.1)	39 (24.4)	40 (23.7)
Erenumab	5 (55.6)	63 (39.4)	68 (40.2)
Eptinezumab	0	4 (2.5)	4 (2.4)
Gepant small molecules			
Ubrogepant	0	14 (8.8)	14 (8.3)
Rimegepant	0	11 (6.9)	11 (6.5)
Combination monoclonal antibodies and Gepants	0	13 (8.1)	13 (7.7)

Abbreviations: CGRP, calcitonin gene-related peptide; NOS, not otherwise specified; RP, Raynaud phenomenon.

^a^ Data are presented as number (percentage) of patients unless otherwise specified.

**Table 2.** Summary of Additional Clinical Characteristics of the Cohort With Complications

Characteristic	Patient 1	Patient 2	Patient 3	Patient 4	Patient 5	Patient 6	Patient 7	Patient 8	Patient 9
Age, y/sex	40s/F	40s/F	50s/F	40s/F	20s/F	30s/F	50s/F	20s/F	30s/F
RP risk factors									
Medications	Amphetamine, β-blocker, triptan	Triptan	Triptan	Triptan	β-Blocker, triptan	β-Blocker, triptan	β-Blocker, triptan	Amphetamine, β-Blocker, triptan	β-Blocker, triptan
Anatomical findings	None	Carpal tunnel syndrome	None	None	None	None	None	None	None
Rheumatologic conditions	Primary RP	Primary RP	Scleroderma, Sjögren syndrome	None	Connective tissue disease, undifferentiated	None	Scleroderma, dermatomyositis	Paraneoplastic pemphigus	None
Vascular risk factors	None	Hypertension, hyperlipidemia	None	None	None	Hypertension	Hypertension	None	None
Migraine history									
Type	Chronic	Chronic	Chronic	Chronic	Chronic	Chronic	Chronic	Episodic	Chronic
Age of diagnosis, y	35	9	13	40	27	Unknown	Unknown	Unknown	Childhood
Frequency (per month)	30	30	30	30	28	>15	16	Unknown	>15
Presence of aura	No	Visual	Visual	No	Visual	No	No	Yes, unspecified	No
Current short-term treatments	Naproxen, prochlorperazine, rizatriptan, hydrocodone	Rizatriptan, sumatriptan, acetaminophen	Butalbital, aspirin, caffeine, codeine, ketotolac, diphenhydramine, prochlorperazine	Butalbital-acetaminophen-caffeine, alprazolam, acetazolamide, cyclobenzaprine,	Naproxen, sumatriptan, promethazine, flurbiprofen	Rizatriptan	Hydrocodone, acetaminophen-caffeine, acetaminophen, ibuprofen, promethazine	Ondansetron, oxycodone	Rizatriptan, acetaminophen-caffeine
Current prophylactic treatments	Galcanezumab, duloxetine	Erenumab, topiramate, gabapentin, onabotulinumtoxin A	Fremanezumab, greater occipital nerve blocks, onabotulinumtoxin A, venlafaxine	Galcanezumab, onabotulinumtoxin A, sodium valproate, lamotrigine, Nerivio Migra	Onabotulinumtoxin A	Erenumab	Bilateral occipital nerve blocks, gabapentin	Onabotulinumtoxin A	Erenumab, magnesium, nortriptyline, topiramate
Past short-term treatments	Naproxen	Naproxen	Rizatriptan, triptan nasal spray, tizanidine	Oxycodone, ondansetron, promethazine, ketorolac, diphenhydramine, hydrocodone-acetaminophen, triptans, dihydroergotamine, ubrogepant	Acetaminophen-caffeine	Zolmitriptan	Naratriptan, rizatriptan, diphenhydramine, hydrocodone-acetaminophen, prochlorperazine, butalbital, tizanidine, lasmiditan, celecoxib	Rizatriptan	Zolmitriptan, prochlorperazine, butalbital-acetaminophen-caffeine, acetaminophen, diphenhydramine, sumatriptan, cyclobenzaprine,
Past prophylactic treatments	Topiramate, sodium valproate, verapamil, onabotulinumtoxin A, occipital nerve block, atenolol, amitriptyline, nortriptyline, gabapentin	Acetazolamide, atenolol, metoprolol	Topiramate, magnesium, vitamin B_2_	Amitriptyline, gamma core, prednisone, occipital nerve block, bilateral C2/3 medial nerve branch block, greater/lesser occipital nerve blocks, vilazodone, pregabalin, gabapentin, trigger point injections including bilateral rhomboids, trapezius muscles, and cervical paraspinals (local only)	Erenumab, verapamil, atenolol	Nadolol	Galcanezumab, erenumab, amitriptyline, duloxetine, pregabalin, propranolol, onabotulinumtoxin A, prednisone, memantine, dexamethasone	Erenumab, galcanezumab, propranolol, gabapentin	Greater occipital nerve blocks, fluoxetine, propranolol, medical massage
CGRP antagonist treatment history									
Medication	Galcanezumab	Erenumab	Fremanezumab	Galcanezumab	Erenumab	Erenumab	Galcanezumab	Erenumab	Erenumab
Dose	120 mg	70 mg	225 mg	120 mg	70 mg	140 mg	120 mg	70 mg	70 mg
Frequency	Every 30 d	Every 30 d	Every 30 d	Every 28 d	Every 30 d	Every 30 d	Every 30 d	Every 30 d	Every 30 d
Total duration of use, mo	18	Unknown	15	12	4	7	12	16	20
RP history									
Duration of RP before starting CGRP antagonist treatment, y	20	2	6	NA (new diagnosis of RP)	27	NA (new diagnosis of RP)	13	NA (new diagnosis of RP)	NA (new diagnosis of RP)
Other rheumatologic conditions	None	Scleroderma	Scleroderma, Sjögren syndrome	None	Connective tissue disease, undifferentiated	None	Scleroderma, dermatomyositis	Paraneoplastic pemphigus	None
Frequency	Daily	Unknown	Daily	Unknown	Unknown	Daily	Daily	Daily	Occasionally
History of RP-associated complications (before CGRP antagonist use)	Peeling of skin on distal digits	None	None	Unknown	None	Blistering, pain	Digital calcinosis	None	None
Complications									
Type	Worsening RP	Worsening RP	Worsening RP	New diagnosis of RP	Worsening RP	New diagnosis of RP	Worsening facial telangiectasias on majority of face and bilateral thumb acro-osteolysis with subcutaneous digital calcinosis	New diagnosis of RP with gangrene and digital autonecrosis that required amputation of left fourth and fifth distal digits	New diagnosis of RP
Duration from onset of CGRP antagonist treatment until onset of complication, d	230	46	127	93	93	26	176	365	314
Outcome	Continued exacerbation (no cessation of galcanezumab therapy)	Resolution (no cessation of erenumab therapy)	Resolution (no cessation of fremanezumab therapy)	Resolution (no cessation therapy of galcanezumab)	Resolution (cessation of erenumab therapy given lack of improvement in migraine management)	Resolution (no cessation of erenumab)	Persistent telangiectasias, currently undergoing laser treatment (cessation of galcanezumab therapy)	Distal phalange amputation (cessation of erenumab therapy)	Continued exacerbation (no cessation of erenumab therapy)
Other possible confounders	Hematoma overlying finger affected by RP		Symptom worsening during wintertime typically						Nadolol treatment (for almost 20 y)

Abbreviations: CGRP, calcitonin gene-related peptide; NA, not applicable; RP, Raynaud phenomenon.

## Discussion

In this cohort study, 5.3% of patients with migraine with underlying or undiagnosed RP who were prescribed CGRP modulators experienced microvascular complications. Comparative analysis of the 2 cohorts of patients (those with complications and those without) did not yield statistically significant differences in demographic characteristics, migraine characteristics, rheumatologic history, RP risk factor history, or CGRP antagonist use (Table 1).

The natural history of primary RP is poorly characterized in the literature, precluding comparison of this study’s complication rate to the historical rates of RP progression to ulceration or infarction. There is no evidence of digital ischemic injury in primary RP.^8,9^ One study^10^ followed up 78 patients with primary RP for 14 years, noting no associated morbidity or progression to scleroderma or other secondary rheumatologic conditions, and concluding that primary RP most commonly remains a benign condition. However, secondary RP frequently leads to vasculopathic damage, including ulceration and infarction.^11^ Approximately 50% of patients with systemic sclerosis will experience digital ulceration at some point in the disease course.^12^ In the patient cohorts in this study, the duration of RP before CGRP antagonist exposure was not correlated with progression to complications because approximately half of the patients with complications (4 of 9 patients) were newly diagnosed with RP after CGRP exposure. However, the possibility that those patients experienced subclinical RP not documented in the medical history cannot be excluded. Of the 2 patients who experienced severe complications, only 1 had a history of RP, a patient with symptomatic systemic sclerosis. Of importance, the small sample size of the cohort with complications in this study (n = 9) limits meaningful commentary about the association between RP duration and complication rate. However, the known natural history of secondary RP (50% prevalence of digital ulceration in patients with systemic sclerosis^12^) suggests that additional caution may be warranted when prescribing CGRP antagonists to patients with secondary RP.

Diminished CGRP activity contributes to RP pathophysiology.^2,3,4,5^ However, clinical trials of the novel CGRP antagonists did not exclude patients with RP. One study^6^ excluded patients with clinically significant cardiovascular disease or vascular ischemia, including peripheral extremity ischemia. Notably, 1 patient in the galcanezumab study^13^ developed a severe flare-up of RP, which subsequently resolved spontaneously without trial withdrawal. To date, prescribing recommendations for all 6 CGRP modulators do not list RP as a contraindication to CGRP antagonist use.

In addition, the co-occurrence of migraine and RP is well established.^14,15,16^ One case-control study^14^ in patients with migraines found that 25% had RP. Another study^17^ reported that 32% of patients with scleroderma had comorbid migraine. Among 9 individuals with RP taking CGRP antagonists who exhibited microvascular complications, 4 (44%) had migraine with aura, which is nearly double the known prevalence of aura in patients with migraine (25%).^18^ Migraine with aura is an established risk factor for cerebrovascular and cardiovascular ischemia, particularly in women.^19^ Further studies are needed to evaluate the microvascular complication in individuals with migraine with aura who are taking CGRP antagonists.

In the current study, 2 patients (1.2%) with RP who were taking CGRP antagonists experienced devastating sequelae. The first patient with serious complications had a history of secondary RP attributable to scleroderma and experienced sudden profusion of facial telangiectasias and accelerated, radiographically demonstrated, digital acro-osteolysis with subcutaneous digital calcinosis (Figure 1). This finding highlights that vasoactive effects of CGRP antagonists may present in diverse ways in the context of scleroderma, which has additional vascular features beyond RP, including telangiectasias. The second patient with severe complications had no preexisting history of RP and experienced digital autonecrosis that required amputation (Figure 2). Digital ulcerations or infarctions rarely occur in primary RP alone and almost always reflect a secondary cause, which may include associated connective tissue diseases or exposure to precipitating medications, such as vasoactive modulators.^20^ This understanding reaffirms concerns about the use of CGRP agents in primary RP, where complicating digital infarctions or ulcerations would ordinarily be highly atypical. These severe adverse events warrant caution when considering the use of these agents in patients with RP, particularly in those with secondary RP.

### Limitations

This study has several limitations. The relatively short duration of follow-up may have limited detection of microvascular complications associated with CGRP antagonist exposure. The study is also limited by data availability. For example, patients with more minor RP exacerbations may not have presented to their physicians or may have presented to outside facilities, limiting the ability to accurately capture microvascular complication rates. To mitigate confounders, an extensive comparative analysis of patient demographic and clinical characteristics was performed, with a particular focus on RP risk factors. Nevertheless, the retrospective nature of the study confers no standardization of exposure, rendering the study vulnerable to confounders. Furthermore, this study was conducted shortly after the US Food and Drug Administration approval and release of CGRP antagonists; thus, a relatively small proportion of the Mayo Clinic’s total patients with migraine were prescribed these medications. The small sample size therefore limits the power of the study. Thus, it cannot be excluded that the lack of statistically significant differences among demographic or clinical characteristics between the 2 cohorts is attributable to limitations arising from the small sample size. In addition, from our experience, patients with severe or medication-refractory migraine were more likely to be prescribed the novel CGRP antagonists, which may limit generalizability of the findings. In addition, RP is a commonly overdiagnosed condition, often deliberately so, to ensure timely identification of patients with early-stage rheumatologic disease.^21^ Potential erroneous inclusion of patients without RP in this analysis could underestimate the microvascular complication rate in patients with true RP who are taking CGRP antagonists. Finally, although temporal associations are drawn between CGRP exposure and RP exacerbations, the observational nature of the study cannot be used to determine causality.

## Conclusions

The results of this study indicate that microvascular complications of CGRP antagonist use in patients with underlying RP are uncommon. No statistically significant differences were found in demographic or clinical characteristics between the 2 patient cohorts (patients taking CGRP antagonists with complications vs those without complications). Specifically, no statistically significant differences were found in risk factors for RP, migraine history, or specific CGRP antagonist medication. Nonetheless, the rare incidence of serious adverse events, including infarction that requires amputation, warrants caution when considering the use of these agents in patients with RP. Future directions include conduction of a retrospective cohort study that examines the microvascular complication rates in patients with and without RP who are taking CGRP antagonists. A hazard ratio extracted from this data would inform the risk profile of CGRP antagonists in this subpopulation.
